# Induction strategies for preventing hemodynamic changes after intubation in non-cardiac surgery patients: a network meta-analysis of randomized controlled trials

**DOI:** 10.3389/fmed.2026.1694700

**Published:** 2026-01-22

**Authors:** Yunfan Gan, Xiaoling Yang, Weiliao Wang, Han Zhang, Xian Luo, Maohua Wang, Zhaojin Xu, Song Su, Jiali Wu

**Affiliations:** 1 Department of Anesthesiology, The Affiliated Hospital of Southwest Medical University, Luzhou, China; 2 Department of General Surgery (Hepatobiliary Surgery), The Affiliated Hospital of Southwest Medical University, Luzhou, China

**Keywords:** general anesthesia, hemodynamics, intubation, network meta-analysis, non-cardiac surgery

## Abstract

**Background:**

Tracheal intubation and laryngoscopy during general anesthesia induce significant hemodynamic changes. Although generally transient, these physiological perturbations may precipitate critical cardiovascular events in high-risk populations. Anesthesiologists have used various drug combinations to suppress this response. This network meta-analysis (NMA) aimed to identify a drug combination that can better suppress hemodynamic fluctuations caused by tracheal intubation in non-cardiac surgical patients.

**Methods:**

We searched 3 different medical literature databases. A NMA was performed on the included randomized controlled trials (RCTs). RCTs were evaluated using the Cochrane risk of bias tool. A random effects network meta-analysis was performed within a frequentist framework. The effects of each pharmacological strategy on intraoperative hemodynamics in patients undergoing non-cardiac surgery were compared. Endpoints included ΔMean Arterial Pressure (ΔMAP) and ΔHeart Rate (ΔHR).

**Results:**

The network meta-analysis included 10 studies and 791 patients. According to the surface under the cumulative ranking curve, Oxycodone-Propofol-Lidocaine (87.4%) demonstrated superior efficacy in controlling fluctuations in MAP, followed by Fentanyl-Propofol-Dexmedetomidine (82.9%) and Fentanyl-Propofol-Clonidine (81.6%). Fen-Pro-Dex (94.8%) demonstrated superior efficacy in controlling fluctuations in HR, followed by Fentanyl-Propofol-Lidocaine (Epidural) (83.3%), Fentanyl-Propofol-Remifentanil (79.1%).

**Conclusion:**

Among patients undergoing non-cardiac surgery, Oxy-Pro-Lid was preferred for attenuating post-intubation changes in MAP, whereas Fen-Pro-Dex provided superior control of HR fluctuations. These findings may help guide the selection of induction pharmacological strategies, although more randomized controlled trials are needed to confirm these results and clarify optimal dosing.

**Systematic Review Registration:**

https://www.crd.york.ac.uk/PROSPERO/view/CRD42024591333, identifier CRD42024591333.

## 1 Introduction

General anesthesia (GA) is the prevailing method of clinical anesthesia and has been widely applied to various types of surgery. As a method of airway management, tracheal intubation plays an essential role in sustaining life during general anesthesia (1). However, during the procedure of laryngoscopy and tracheal intubation, mechanical stimulation can activate the cervical sympathetic nerves, leading to increased blood pressure and tachycardia (2, 3). These cardiovascular reflexes potentially generate some life-threatening complications, such as myocardial ischemia and cerebral hemorrhage, which pose a significant challenge for anesthesiologists (2, 4–6). Therefore, anesthesiologists normally administer various types of medications during induction to attenuate the hemodynamic changes induced by tracheal intubation.

Currently, various types of anesthetic drugs are used for induction, and anesthesiologists typically exhibit variations in their medication preferences. Given the numerous medication options, it is necessary to conduct further research in this field to identify the most suitable combination of drugs to alleviate cardiovascular reflexes caused by tracheal intubation. Although there have been numerous studies (7–9) exploring strategies for maintaining hemodynamic stability after anesthesia induction, most of this research is limited to comparisons of only two or three drug strategies. The comparative efficacy of various drug combinations has not yet been comprehensively assessed, posing a challenge for anesthesiologists in determining the optimal pharmacological approach.

Previous studies on intubation-related hemodynamic fluctuations have primarily focused on cardiac surgery. However, the cardiovascular responses to laryngoscopy and intubation are not limited to cardiac surgery and also occur during non-cardiac procedures, where they may affect perioperative outcomes. In addition, induction strategies in this population may receive less attention. Therefore, there is a need for a comprehensive comparison of induction strategies in non-cardiac surgery.

To provide comparative evidence, this study conducts a comprehensive systematic review of all currently available anesthesia induction pharmacological strategies applied in non-cardiac surgeries and evaluates their relative effects via network meta-analysis.

## 2 Materials and methods

### 2.1 Study registration

This network meta-analysis adhered to the Preferred Reporting Items for Systematic Reviews and Meta-Analyses (PRISMA) guidelines. The study protocol was registered with the International Prospective Register of Systematic Reviews (PROSPERO) under registration number CRD42024591333.

### 2.2 Search strategy

We searched all articles published in PubMed, Embase, and the Cochrane Library databases from database inception to 31 December 2024. The combination of subject words and free words was utilized, incorporating the specified MeSH terms: “hemodynamics,” “Blood Pressure,” “Heart Rate,” “Intubation,” “Intratracheal,” “Anesthesia,” “randomized.” The inclusion criteria were limited to randomized controlled trial studies only.

### 2.3 Selection criteria

The study follows the inclusion criteria: (1) the articles are randomized controlled trials (RCTs); (2) the anesthesia method used is general anesthesia, including elective surgery or emergency surgery; (3) the subjects consist of all ages; (4) the endpoint of the study include mean arterial pressure (MAP) and heart rate (HR). The exclusion criteria were: (1) the type of operation is cardiac surgery; (2) non-English articles, letters, reviews, case reports, non-human studies; (3) Non-RCTs or single-arm studies; (4) inability to obtain outcome data.

This NMA included RCTs that compared at least two pharmacological strategies. The PICO-SD details are provided below:

1. Patients (P): Patients of all ages undergoing elective or emergency general anesthesia, excluding those who underwent cardiac surgery.
2. Intervention (I): Pharmacological strategy of general anesthesia induction.
3. Comparison (C): Other pharmacological strategies.
4. Outcome measurements (O): ΔMAP and ΔHR were defined as the differences between the maximum MAP and HR values recorded from post-intubation to pre-incision and their respective baseline values. Baseline values were taken as the measurements obtained when the patient was at rest prior to induction of anesthesia.
5. Study design (SD): We included RCTs published in English between database inception and 2024. Review articles, case reports, case series, and other non-relevant studies were excluded.

### 2.4 Data extraction and quality assessment

Two researchers independently extracted data, and the following information was recorded in a Microsoft Excel spreadsheet: title; first author; publication year; country; type of surgery; age; ASA grade; inclusion criteria; exclusion criteria; sample size for each group; pharmacological strategy for induction; hemodynamic data (from different periods).

Data were extracted from tables or text. If specific values were not presented in the chart, Plot Digitizer (version 1.3) was utilized to extract the data from the result report graph. For multi-arm trials with the same drug combination but different doses, we pooled the arms representing the same intervention into a single group. We used sample sizes, medians, interquartile ranges, standard errors, and 95% confidence intervals to calculate the missing data, as suggested by the Cochrane Handbook guidelines.

The quality of all included studies was independently assessed by the two researchers using the Cochrane Risk of Bias Tool (RoB 1.0) for Randomized Controlled Trials. This scale was used to assess six domains of bias (low, high, or unclear) for each included study: selection bias, performance bias, detection bias, attrition bias, reporting bias, and other bias (10). Any discrepancies arising during the evaluation process were resolved by discussion between the two reviewers or mediation by a third reviewer.

### 2.5 Statistical analysis

The endpoints of this NMA were ΔMAP and ΔHR (relevant definitions provided in the section “2.3 Selection Criteria”). The hemodynamic data (MAP, HR) were presented as mean ± standard deviation.

When multiple pharmacological strategies form an evidence network, a network evidence plot was created. Each pharmacological strategy was treated as a node in this network. The size of the node represents the sample size, while the width of the line is proportional to the number of trials for the assessment comparison.

Contribution plots were used to represent the contributions of direct versus indirect comparisons of included studies to the final results.

Local inconsistency was used to assess network consistency. Given the heterogeneity among studies, a random-effects consistency model was employed for data analysis.

Forest plots were used to compare the relative treatment effects between the two strategies, and the results are presented as mean differences with 95% confidence intervals.

The frequentist method was utilized to estimate the overall ranking of pharmacological strategies in NMA by calculating the probability of ranking for each strategy. The surface under the cumulative ranking curve (SUCRA) was employed to visually represent the overall ranking of different induction strategies for inhibiting hemodynamic fluctuations during tracheal intubation. SUCRA values range from 0% to 100%. A higher SUCRA value indicates superior ranking of pharmacological strategies, suggesting better inhibition of the cardiovascular reflex after tracheal intubation (11).

All network meta-analyses were performed using Stata 17.0 (StataCorp, College Station, TX, USA). Using “network” package to create network plots and perform local inconsistency tests, and “mvmeta” package for the frequentist random-effects network meta-analysis. A two-tailed *p*-value < 0.05 was considered statistically significant. All statistical methods were consulted with a statistics expert.

## 3 Results

### 3.1 Study selection and characteristics

We searched a total of 2715 records in Pubmed, Cochrane Library, and Embase databases. After removing 516 duplicates, we screened the titles and abstracts, resulting in 86 remaining articles. Following a full-text review, 76 articles were excluded based on the exclusion criteria, leaving 10 articles for further analysis.

**FIGURE 1:**
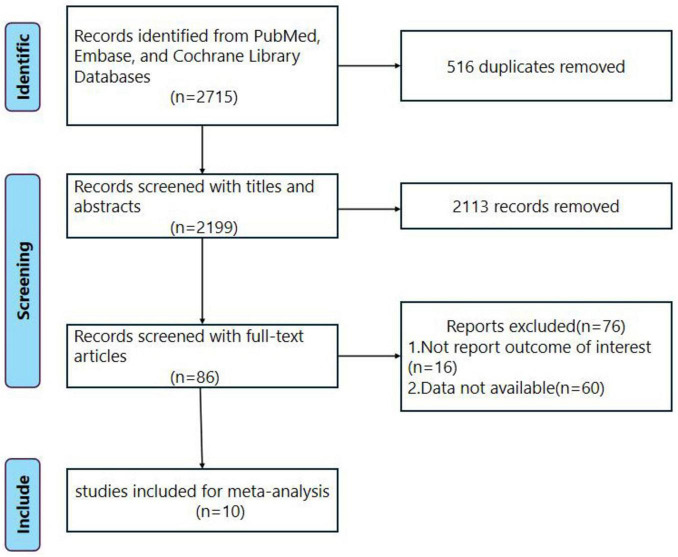
Flowchart of trial inclusion and exclusion.

The flowchart depicting the literature screening process is presented in Figure 1, while Table 1 outlines the basic characteristics of the 10 RCTs included in the NMA. Studies were conducted in Egypt (12), Thailand (13), East Africa (6), China (3, 14), Iran (15), Korea (16), India (17) and Japan (2, 18).

**TABLE 1:** Basic characteristics of randomized controlled trials included in the network meta-analysis.

References	Country	Type of surgery	Age	ASA	GA protocol	Focused strategies	Dosage	Sample size	End point
Amin et al. (12)	Egypt	Non-cardiac	>60	I-III	Propofol + Rocuronium + Lidocaine	Propofol + Lidocaine	1–1.5 mg/kg + 1 mg/kg	47	ΔMAP ΔHR
					Propofol + Rocuronium + Fentanyl	Propofol + Fentanyl	1–1.5 mg/kg+1 mcg/kg	46	
Seangrung et al. (13)	Thailand	Non-cardiac	18–65	I-II	Propofol + Fentanyl + Rocuronium + Dexmedetomidine	Propofol + Fentanyl + Dexmedetomidine	1.5 mg/kg+1 mcg/ kg+1 mcg/kg	53	ΔMAP ΔHR
					Propofol + Fentanyl + Rocuronium + Lidocaine	Propofol + Fentanyl + Lidocaine	2.0 mg/kg + 1 mcg/ kg + 1 mg/kg	53	
Ongewe et al. (6)	East Africa	East Africa	Non-cardiac	I-II	Propofol + Cisatracurium + Fentanyl	Propofol + Fentanyl	2 mg/kg + 0.5 mg/kg	54	ΔMAP ΔHR
					Propofol + Cisatracurium + Ketamine	Propofol + Ketamine	2 mg/kg + 0.5 mg/kg	54	
Pan et al. (14)	China	Hepatic	18–65	I-II	Propofol + Fentanyl + Cisatracurium + Dexmedetomidine	Propofol + Fentanyl + Dexmedetomidine	1.5 mg/kg + 3 mcg/kg + 0.4 mcg/kg	47	ΔMAP ΔHR
					Propofol + Fentanyl + Cisatracurium + Remifentanil	Propofol + Fentanyl + Remifentanil	1.5 mg/kg + 3 mcg/kg + 3 μg/kg/h	47	
Nasseri et al. (15)	Iran	Abdominal	N/A	I-II	Propofol + Fentanyl + Succinylcholine + Remifentanil	Propofol + Fentanyl + Remifentanil	2 mg/kg + 1 mcg/kg + 1 mcg/kg	29	ΔMAP ΔHR
					Propofol + Fentanyl + Succinylcholine + NS	Propofol + Fentany	2 mg/kg + 1 mcg/kg	30	
Kakkar et al. (17)	India	Non-cardiac	18–60	I-II	Propofol + Fentanyl + Vecuronium + Dexmedetomidine	Propofol + Fentanyl + Dexmedetomidine	2–2.5 mg/kg + 1 mcg/ kg + 0.5(1) μg/kg	30 + 30	ΔMAP ΔHR
					Propofol + Fentanyl + Vecuronium + Clonidine	Propofol + Fentanyl + Clonidine	2–2.5 mg/kg + 1 mcg/ kg + 1 mcg/kg	30	
Yang et al. (3)	China	Thoracic	22–60	I-II	Propofol + Fentanyl + Vecuronium + NS(Epidural)	Propofol + Fentany	3.5 mcg/mL(TCI) + 3 mcg/kg	20	ΔMAP ΔHR
					Propofol + Fentanyl + Vecuronium + Lidocaine	Propofol + Fentanyl + Lidocaine	3.5 mcg/mL(TCI) + 3 mcg/kg + 2 mg/kg	20	
Sawano et al. (2)	Japan	General/urologic/ gynecological	N/A	I-II	Propofol + Rocuronium + NS	Propofol	4 mcg/mL(TCI) + 2(4) mg/kg	30	ΔMAP ΔHR
					Propofol + Rocuronium + Fentanyl	Propofol + Fentany	3 mg/kg + 2 μg/kg	30 + 30	
Miyazaki et al. (18)	Japan	General/urologic/ gynecological	N/A	I-II	Propofol + Vecuronium + Fentanyl + Landiolol	Propofol + Fentanyl + Landiolol	2 mg/kg + 2 μg/kg + 0.1 mg/kg	9	ΔHR
					Propofol + Vecuronium + Fentanyl + Nicardipine	Propofol + Fentanyl + Nicardipine	2 mg/kg + 2 μg/ kg + 1 mg	9	
Lee et al. (16)	Korea	Nasal	20–65	I-II	Lidocaine + Propofol + Succinylcholine + Fentanyl	Propofol + Fentanyl + Lidocaine	2 mg/kg + 2 mcg/ kg + 40 mg	32	ΔMAP ΔHR
					Lidocaine + Propofol + Succinylcholine + Oxycodone	Ropofol + Oxycodone + Lidocaine	2 mg/kg + 0.2 mg/ kg + 40 mg	32	

Focused strategies refer to drugs known to effectively suppress hemodynamic changes after laryngoscopy and tracheal intubation. In some studies, there may be multiple subgroups of the same drug combination. No., number of patients.

### 3.2 Risk of bias assessment

**FIGURE 2:**
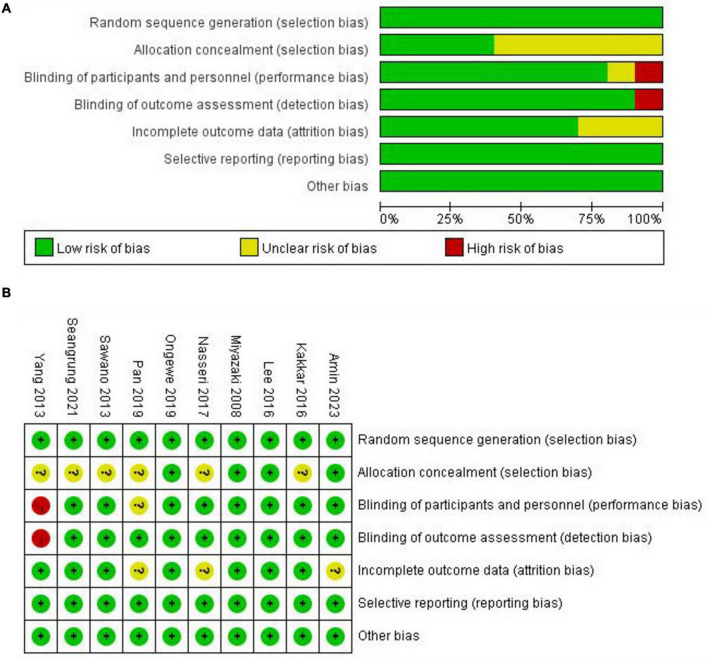
Cochrane Collaboration’s risk of bias assessment. **(A)** Risk of bias graph representing the assessment of each risk of bias item, presented as percentages within all included studies. **(B)** Risk of bias summary for each included studies.

The risk of bias of the included studies was assessed using the Cochrane Risk of Bias tool, as presented in Figure 2.

### 3.3 Network meta-analysis results for ΔMAP and ΔHR

**FIGURE 3:**
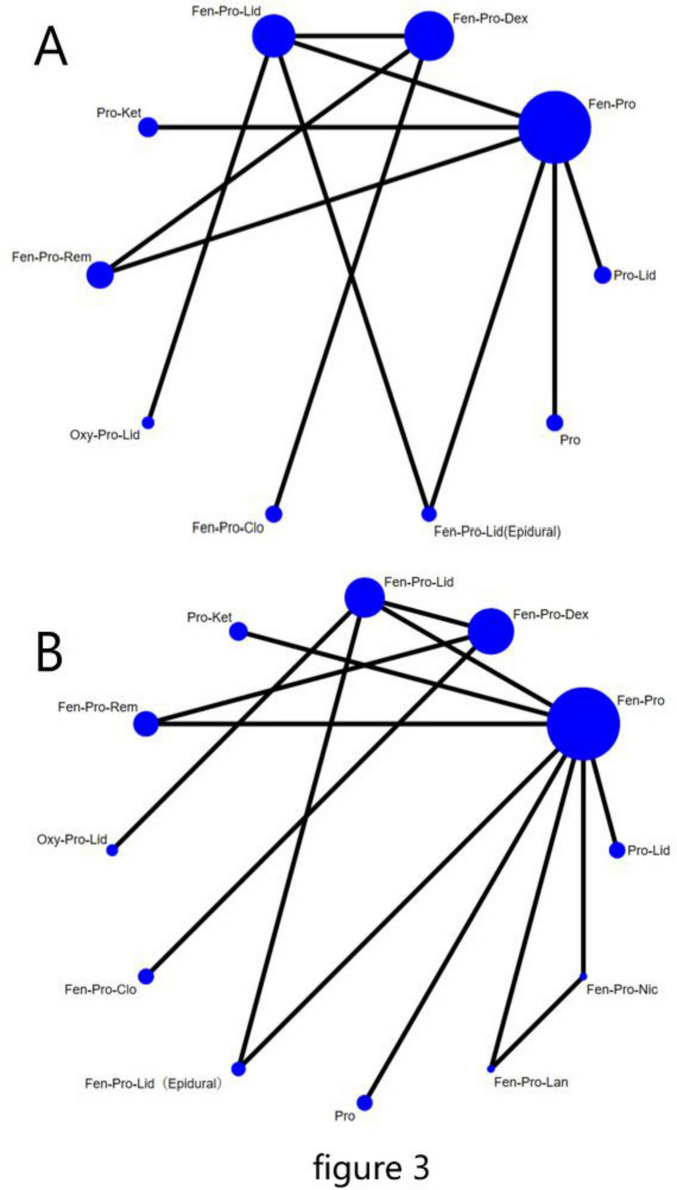
Network plot of all the included studies. The nodes represent different pharmacological combinations for preventing hemodynamic changes after laryngoscopy and endotracheal intubation in patients undergoing non-cardiac surgery, and the edges show the direct comparisons available between them. The nodes and edges are weighted by the number of patients included and the standard error of the mean difference, respectively. **(A)** ΔMAP, **(B)** ΔHR. Fen, Fentanyl; Pro, Propofol; Dex, Dexmedetomidine; Lid, Lidocaine; Ket, Ketamine; Rem, Remifentanil; Oxy, Oxycodone; Clo, Clonidine; Lid(Epidural), Lidocaine(Epidural); Lan, landiolol; Nic, Nicardipine.

**FIGURE 4:**
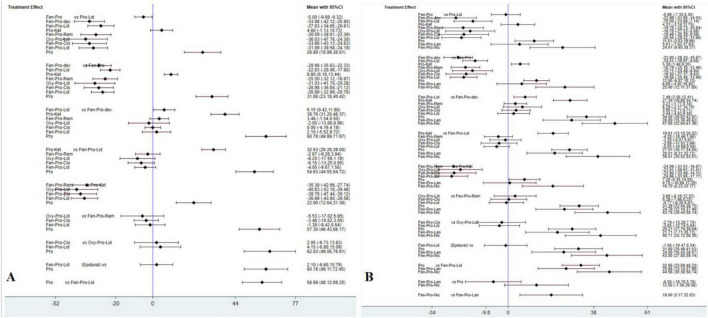
Confidence interval plots between each pharmacological strategies. The diamond shape represents the mean summary effects; the black line, 95% CI. **(A)** ΔMAP; **(B)** ΔHR.

For all endpoints, we presented the network plot (Figure 3), Interval plot (Figure 4). Contribution plots, inconsistency plots, and SUCRA values for each pharmacological strategy are provided in Supplementary Figures 1–3, respectively.

For ΔMAP, a total of 10 pharmacological strategies from 9 studies were compared; for ΔHR, a total of 12 pharmacological strategies from 10 studies were compared. Figures 3A, B display the network plots illustrating the sample size and direct and indirect comparisons of each pharmacological strategy. For both endpoints, Fentanyl-Propofol (Fen-Pro) was the most frequently compared drug combination, followed by Fentanyl-Propofol-Dexmedetomidine (Fen-Pro-Dex) and Fentanyl-Propofol-Lidocaine. (Fen-Pro-Lid) No statistical differences were found in inconsistency testing for either endpoint (Supplementary Figure 2). Figure 3 provides the abbreviations for all pharmacological strategies.

The comparison of ΔMAP of each strategy obtained from NMA is presented in Figure 4A, where Oxycodone-Propofol-Lidocaine (Oxy-Pro-Lid) showed lowest ΔMAP compared to the other nine pharmacological strategies. The rankogram and cumulative ranking plot (Supplementary Figure 3A) showed that Oxy-Pro-Lid had the lowest ΔMAP. The top three SUCRA rankings are: Oxy-Pro-Lid (87.4%), Fen-Pro-Dex (82.9%), and Fentanyl-Propofol-Clonidine (Fen-Pro-Clo) (81.6%). The SUCRA results revealed that Oxy-Pro-Lid was the top-ranked pharmacological strategy for mitigating mean arterial pressure fluctuations after intubation.

The comparison of ΔHR of each strategy obtained from NMA is shown in Figure 4B, which Fen-Pro-Dex showed lowest ΔHR compared to the other 11 pharmacological strategies. The rankogram and cumulative ranking plot (Supplementary Figure 3B) showed that Fen-Pro-Dex had the lowest ΔHR. The top three SUCRA rankings are: Fen-Pro-Dex (94.8%), Fen-Pro-Lid(Epidural) (83.3%), and Fentanyl-Propofol-Remifentanil (Fen-Pro-Rem) (79.1%). The SUCRA results revealed that Fen-Pro-Dex was the highest-ranked pharmacological strategy for mitigating heart rate fluctuations following intubation.

## 4 Discussion

Attenuating intubation-related hemodynamic responses remains a key goal during anesthetic induction for non-cardiac surgery. This study conducted a network meta-analysis of anesthesia induction protocols for non-cardiac surgery. The results indicated that Oxy-Pro-Lid and Fen-Pro-Dex were the preferred pharmacological strategies for controlling MAP and HR after tracheal intubation, respectively.

It is important to maintain MAP and HR stability during anesthesia induction and tracheal intubation, as this is one of the primary tasks for anesthesiologists. The mechanical stimulation caused by laryngoscopy and tracheal intubation, may lead to increases in MAP and HR, and even result in myocardial ischemia, arrhythmias, and other complications (19, 20). MAP and HR, as important vital signs, are widely used to assess cardiovascular status during anesthesia (15). The studies included in this network meta-analysis evaluated the efficacy of different induction regimens by assessing fluctuations in MAP and HR.

ΔMean Arterial Pressure is defined as the difference between the maximal MAP values recorded from post-intubation to pre-incision and the baseline MAP. This study compared ten different pharmacological strategies. Among these pharmacological strategies, Pro-Lid-Oxy was the highest-ranked pharmacological strategy for inhibiting the increase in MAP after tracheal intubation. Propofol, the most commonly used sedative, rapidly achieves deep sedation by traversing the blood-brain barrier (21). Lidocaine is a local anesthetic and class IB antiarrhythmic drug. Qi et al. (22) proved that intravenous lidocaine alleviates cardiovascular responses induced by laryngoscopy and tracheal intubation. This may be because intravenous lidocaine can increase the threshold of airway stimulation and directly suppress hemodynamic responses (23, 24). Oxycodone activates μ-opioid and κ-opioid receptors and is frequently prescribed for managing moderate to severe pain (25, 26). Oxycodone typically takes effect within 2–3 min after intravenous administration (16). Therefore, it can be effectively used to attenuate the cardiovascular reflex induced by tracheal intubation. Koch et al. (27) indicated that oxycodone provides superior analgesic effects compared to fentanyl but also has more side effects. However, this discrepancy may be attributed to the non-equivalence of their doses, thus requiring cautious consideration when using this medication. Pro-Lid-Oxy, the prioritized drug combination for controlling MAP fluctuations after intubation found in this NMA, may be more suitable for anesthesia induction in patients who need to avoid severe blood pressure fluctuations.

In addition, ΔHR, defined as the difference between the maximal HR values recorded from post-intubation to pre-incision and the baseline HR, was included in the evaluation of twelve different pharmacological strategies. Different from the results obtained by ΔMAP, Fen-Pro-Dex was most effective in inhibiting heart rate fluctuations after laryngoscopy and tracheal intubation. Fentanyl is an opioid analgesic administered in combination with other medications to mitigate adrenergic stress resulting from surgical stimulation (28, 29). Dexmedetomidine, a potent α2 -adrenoceptor agonist, can reduce norepinephrine release and inhibit sympathetic nerve excitability, leading to decreases in heart rate and blood pressure when administered by intravenous injection (13, 30, 31). Therefore, the combined use of fentanyl, dexmedetomidine, and propofol could effectively alleviate the tachycardia caused by laryngoscopy and intubation. Heart rate plays a crucial role in determining myocardial oxygen consumption, and tachycardia significantly elevates myocardial oxygen demand (5). Adrenergic stress can elevate heart rate, leading to adverse events such as myocardial ischemia, heart failure, and tachyarrhythmia. Although the hemodynamic response to laryngoscopy and tracheal intubation is transient in most patients, for those with a history of cardiac disease, this inappropriate response may increase perioperative morbidity and mortality (6, 24). Fen-Pro-Dex, the better combination for controlling heart rate, may be more appropriate for anesthesia induction in patients with ischemic heart disease.

This study has some limitations. First, in the pilot study, the endpoints also included systolic blood pressure and diastolic blood pressure, but it was difficult to construct a satisfactory network evidence plot because of the small number of included studies. Besides, due to the limited number of studies included, the dosage groupings for each pharmacological strategy have not been thoroughly explored. Future study should include more literature and focus on dose-dependent effects. In addition, this study focused solely on drugs that significantly impact hemodynamics and ignored the possible effects of muscle relaxants.

## 5 Conclusion

Significant fluctuations in blood pressure and heart rate during laryngoscopy and tracheal intubation may increase the risk of adverse cardiovascular events in patients undergoing non-cardiac surgery, particularly in those with cardiovascular comorbidity. Our network meta-analysis suggested that Oxy–Pro–Lid was a preferred regimen for attenuating changes in mean arterial pressure, whereas Fen–Pro–Dex showed superior control of heart rate fluctuations. However, future study should incorporate more randomized controlled trials to clarify optimal dosing and safety.

## Data Availability

The raw data supporting the conclusions of this article will be made available by the authors, without undue reservation.
