# Supplementary material for: Induction strategies for preventing hemodynamic changes after intubation in non-cardiac surgery patients: a network meta-analysis of randomized controlled trials
Source: Front Med (Lausanne). 2026 Jan 22;12:1694700. doi: 10.3389/fmed.2026.1694700 (PMC12872843; doi:10.3389/fmed.2026.1694700)
Supplement: Supplementary file 1 [file Supplementary_file_1.docx]

***Supplementary Material***

**PRISMA Checklist**

| **Section and Topic** | **Item #** | **Checklist item** | **Location where item is reported** |
| --- | --- | --- | --- |
| **TITLE** | | |  |
| **Title** | **1** | **Identify the report as a systematic review.** | **Title** |
| **ABSTRACT** | | |  |
| **Abstract** | **2** | **Provide a structured summary including, as applicable: Background: main objectives Methods: data sources; study eligibility criteria, participants, and interventions; study appraisal; and synthesis methods, such as network meta-analysis. Results: number of studies and participants identified; summary estimates with corresponding confidence/credible intervals. Discussion/Conclusions: limitations; conclusions and implications of findings. Other: primary source of funding; systematic review registration number with registry name.** | **Abstract** |
| **INTRODUCTION** | | |  |
| **Rationale** | **3** | **Describe the rationale for the review in the context of existing knowledge.** | **Introduction** |
| **Objectives** | **4** | **Provide an explicit statement of the objective(s) or question(s) the review addresses.** | **Introduction** |
| **METHODS** | | |  |
| **Eligibility criteria** | **5** | **Specify the inclusion and exclusion criteria for the review and how studies were grouped for the syntheses.** | **Materials and Methods: 2.3 Selection Criteria** |
| **Information sources** | **6** | **Specify all databases, registers, websites, organisations, reference lists and other sources searched or consulted to identify studies. Specify the date when each source was last searched or consulted.** | **Materials and Methods: 2.3 Selection Criteria** |
| **Search strategy** | **7** | **Present the full search strategies for all databases, registers and websites, including any filters and limits used.** | **Materials and Methods: 2.3 Selection Criteria** |
| **Selection process** | **8** | **Specify the methods used to decide whether a study met the inclusion criteria of the review, including how many reviewers screened each record and each report retrieved, whether they worked independently, and if applicable, details of automation tools used in the process.** | **Materials and Methods: 2.4 Data Extraction and Quality Assessment** |
| **Data collection process** | **9** | **Specify the methods used to collect data from reports, including how many reviewers collected data from each report, whether they worked independently, any processes for obtaining or confirming data from study investigators, and if applicable, details of automation tools used in the process.** | **Materials and Methods: 2.4 Data Extraction and Quality Assessment** |
| **Data items** | **10a** | **List and define all outcomes for which data were sought. Specify whether all results that were compatible with each outcome domain in each study were sought (e.g. for all measures, time points, analyses), and if not, the methods used to decide which results to collect.** | **Materials and Methods: 2.3 Selection Criteria (Outcome measurements); Materials and Methods: 2.4 Data Extraction and Quality Assessment** |
|  | **10b** | **List and define all other variables for which data were sought (e.g. participant and intervention characteristics, funding sources). Describe any assumptions made about any missing or unclear information.** | **Materials and Methods: 2.4 Data Extraction and Quality Assessment** |
| **Study risk of bias assessment** | **11** | **Specify the methods used to assess risk of bias in the included studies, including details of the tool(s) used, how many reviewers assessed each study and whether they worked independently, and if applicable, details of automation tools used in the process.** | **Materials and Methods: 2.4 Data Extraction and Quality Assessment** |
| **Effect measures** | **12** | **Specify for each outcome the effect measure(s) (e.g. risk ratio, mean difference) used in the synthesis or presentation of results.** | **Materials and Methods: 2.5 Statistical Analysis** |
| **Synthesis methods** | **13a** | **Describe the processes used to decide which studies were eligible for each synthesis (e.g. tabulating the study intervention characteristics and comparing against the planned groups for each synthesis (item #5)).** | **Materials and Methods: 2.5 Statistical Analysis** |
|  | **13b** | **Describe any methods required to prepare the data for presentation or synthesis, such as handling of missing summary statistics, or data conversions.** | **Materials and Methods: 2.5 Statistical Analysis** |
|  | **13c** | **Describe any methods used to tabulate or visually display results of individual studies and syntheses.** | **Materials and Methods: 2.5 Statistical Analysis** |
|  | **13d** | **Describe any methods used to synthesize results and provide a rationale for the choice(s). If meta-analysis was performed, describe the model(s), method(s) to identify the presence and extent of statistical heterogeneity, and software package(s) used.** | **Materials and Methods: 2.5 Statistical Analysis** |
|  | **13e** | **Describe any methods used to explore possible causes of heterogeneity among study results (e.g. subgroup analysis, meta-regression).** | **Materials and Methods: 2.5 Statistical Analysis** |
|  | **13f** | **Describe any sensitivity analyses conducted to assess robustness of the synthesized results.** | **not apply** |
| **Reporting bias assessment** | **14** | **Describe any methods used to assess risk of bias due to missing results in a synthesis (arising from reporting biases).** | **not apply** |
| **Certainty assessment** | **15** | **Describe any methods used to assess certainty (or confidence) in the body of evidence for an outcome.** | **Materials and Methods: 2.5 Statistical Analysis** |
| **RESULTS** | | |  |
| **Study selection** | **16a** | **Describe the results of the search and selection process, from the number of records identified in the search to the number of studies included in the review, ideally using a flow diagram.** | **Results: 3.1 Study Selection and Characteristics; Figure 1** |
|  | **16b** | **Cite studies that might appear to meet the inclusion criteria, but which were excluded, and explain why they were excluded.** | **Figure1** |
| **Study characteristics** | **17** | **Cite each included study and present its characteristics.** | **Results: 3.1 Study Selection and Characteristics; Figure 1** |
| **Risk of bias in studies** | **18** | **Present assessments of risk of bias for each included study.** | **Results: 3.2 Risk of Bias Assessment; Figure 2** |
| **Results of individual studies** | **19** | **For all outcomes, present, for each study: (a) summary statistics for each group (where appropriate) and (b) an effect estimate and its precision (e.g. confidence/credible interval), ideally using structured tables or plots.** | **Results: 3.3 Network Meta-Analysis Results for ΔMAP and ΔHR; Figure 4** |
| **Results of syntheses** | **20a** | **For each synthesis, briefly summarise the characteristics and risk of bias among contributing studies.** | **Results: 3.2 Risk of Bias Assessment; Figure 2; Supplementary Figure S1** |
|  | **20b** | **Present results of all statistical syntheses conducted. If meta-analysis was done, present for each the summary estimate and its precision (e.g. confidence/credible interval) and measures of statistical heterogeneity. If comparing groups, describe the direction of the effect.** | **Results: 3.3 Network Meta-Analysis Results for ΔMAP and ΔHR** |
|  | **20c** | **Present results of all investigations of possible causes of heterogeneity among study results.** | **Supplementary figure S2** |
|  | **20d** | **Present results of all sensitivity analyses conducted to assess the robustness of the synthesized results.** | **not apply** |
| **Reporting biases** | **21** | **Present assessments of risk of bias due to missing results (arising from reporting biases) for each synthesis assessed.** | **Results: 3.2 Risk of Bias Assessment; Figure 2** |
| **Certainty of evidence** | **22** | **Present assessments of certainty (or confidence) in the body of evidence for each outcome assessed.** | **Results: 3.3 Network Meta-Analysis Results for ΔMAP and ΔHR; Figure 4** |
| **DISCUSSION** | | |  |
| **Discussion** | **23a** | **Provide a general interpretation of the results in the context of other evidence.** | **Discussion** |
|  | **23b** | **Discuss any limitations of the evidence included in the review.** | **Discussion** |
|  | **23c** | **Discuss any limitations of the review processes used.** | **not apply** |
|  | **23d** | **Discuss implications of the results for practice, policy, and future research.** | **Discussion** |
| **OTHER INFORMATION** | | |  |
| **Registration and protocol** | **24a** | **Provide registration information for the review, including register name and registration number, or state that the review was not registered.** | **Materials and Methods: 2.1 Study registration (PROSPERO CRD42024591333)** |
|  | **24b** | **Indicate where the review protocol can be accessed, or state that a protocol was not prepared.** | **Materials and Methods: 2.1 Study registration (PROSPERO CRD42024591333)** |
|  | **24c** | **Describe and explain any amendments to information provided at registration or in the protocol.** | **not apply** |
| **Support** | **25** | **Describe sources of financial or non-financial support for the review, and the role of the funders or sponsors in the review.** | **Declarations: Funding** |
| **Competing interests** | **26** | **Declare any competing interests of review authors.** | **Declarations: Competing interests** |
| **Availability of data, code and other materials** | **27** | **Report which of the following are publicly available and where they can be found: template data collection forms; data extracted from included studies; data used for all analyses; analytic code; any other materials used in the review.** | **Declarations: Availability of data and materials** |

**Supplementary figure S1. Contribution plot for each endpoints.** The size of the squares compared for each treatment is proportional to the weight of each summary effect. 01, 02, 03, 04, 05, 06, 07, 08, 09, 10, 11, and 12 correspond to Pro-Lid, Fen-Pro, Fen-Pro-dex, Fen-Pro-Lid, Pro-Ket, Fen-Pro-Rem, Oxy-Pro-Lid, Fen-Pro-Clo, Fen-Pro-Lid (Epidural), Pro, Fen-Pro-Lan, and Fen-Pro-Nic, respectively. A)ΔMAP; B)ΔHR.

**Supplementary figure S2. Inconsistency plots for each endpoints.** Global inconsistency analysis and local inconsistency analysis were used to examine the inconsistency of the included studies. 01, 02, 03, 04, 05, 06, 07, 08, 09, 10, 11, and 12 correspond to Pro-Lid, Fen-Pro, Fen-Pro-dex, Fen-Pro-Lid, Pro-Ket, Fen-Pro-Rem, Oxy-Pro-Lid, Fen-Pro-Clo, Fen-Pro-Lid (Epidural), Pro, Fen-Pro-Lan, and Fen-Pro-Nic, respectively. P < 0.05 indicates that there is inconsistency in the included studies. A), C): Inconsistency test and node-splitting method for ΔMAP; B), D): Inconsistency test and node-splitting method for ΔHR.

**Supplementary figure S3. Surface under the cumulative ranking curve analysis (SUCRA) graphs and specific values** **for each endpoints.** The closer the cumulative ranking curve (SUCRA) value is to 100%, the higher the treatment ranks relative to all other treatments.A), C): SUCRA graphs and the specific values for ΔMAP; B), D): SUCRA graphs and the specific values for ΔHR.
