# Supplementary figures and images for: Induction strategies for preventing hemodynamic changes after intubation in non-cardiac surgery patients: a network meta-analysis of randomized controlled trials
Source: Front Med (Lausanne). 2026 Jan 22;12:1694700. doi: 10.3389/fmed.2026.1694700 (PMC12872843; doi:10.3389/fmed.2026.1694700)

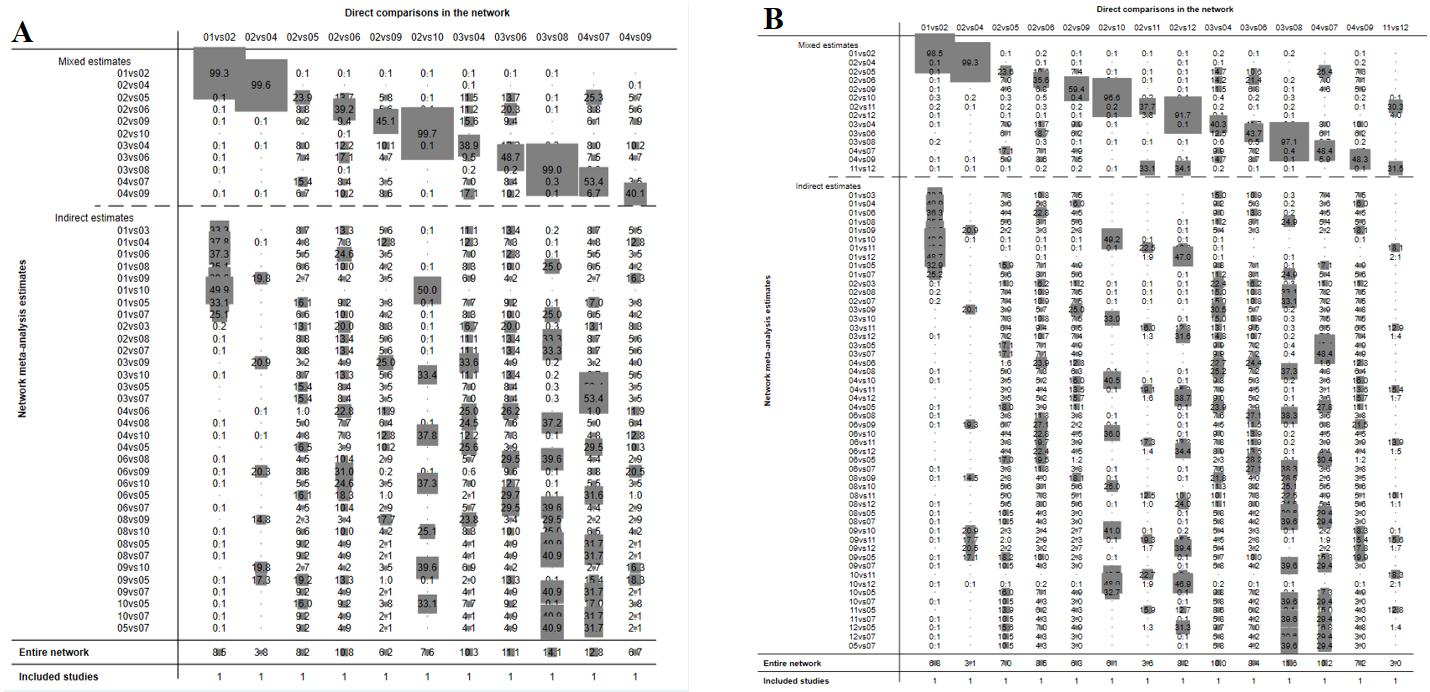

Supplement: Supplementary file 2 [file Image_1.jpg]

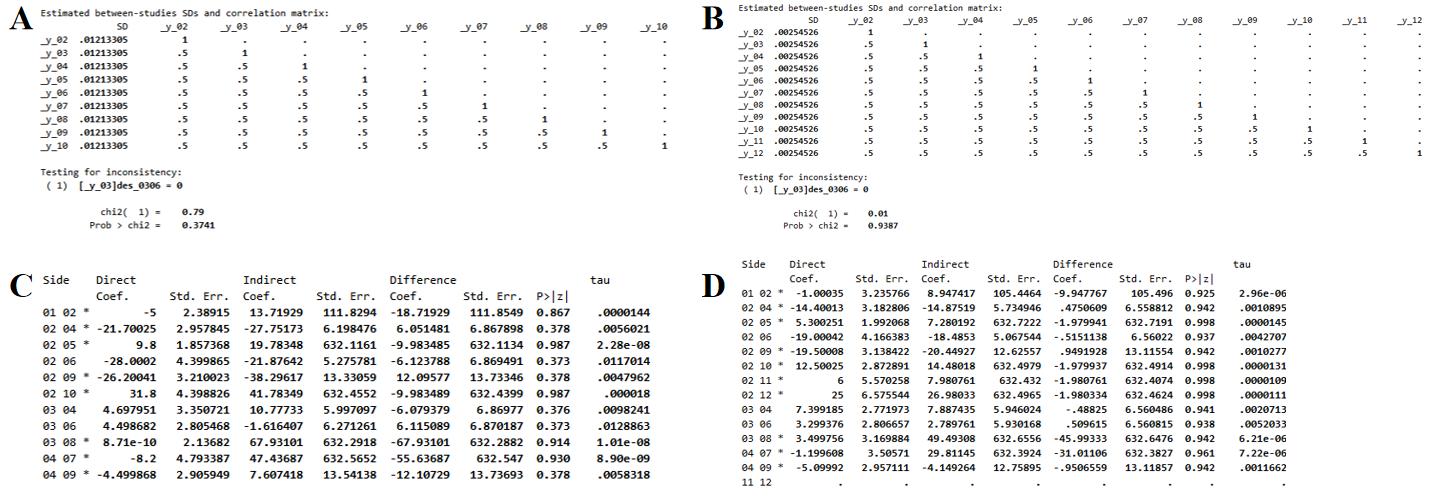

Supplement: Supplementary file 3 [file Image_2.jpg]

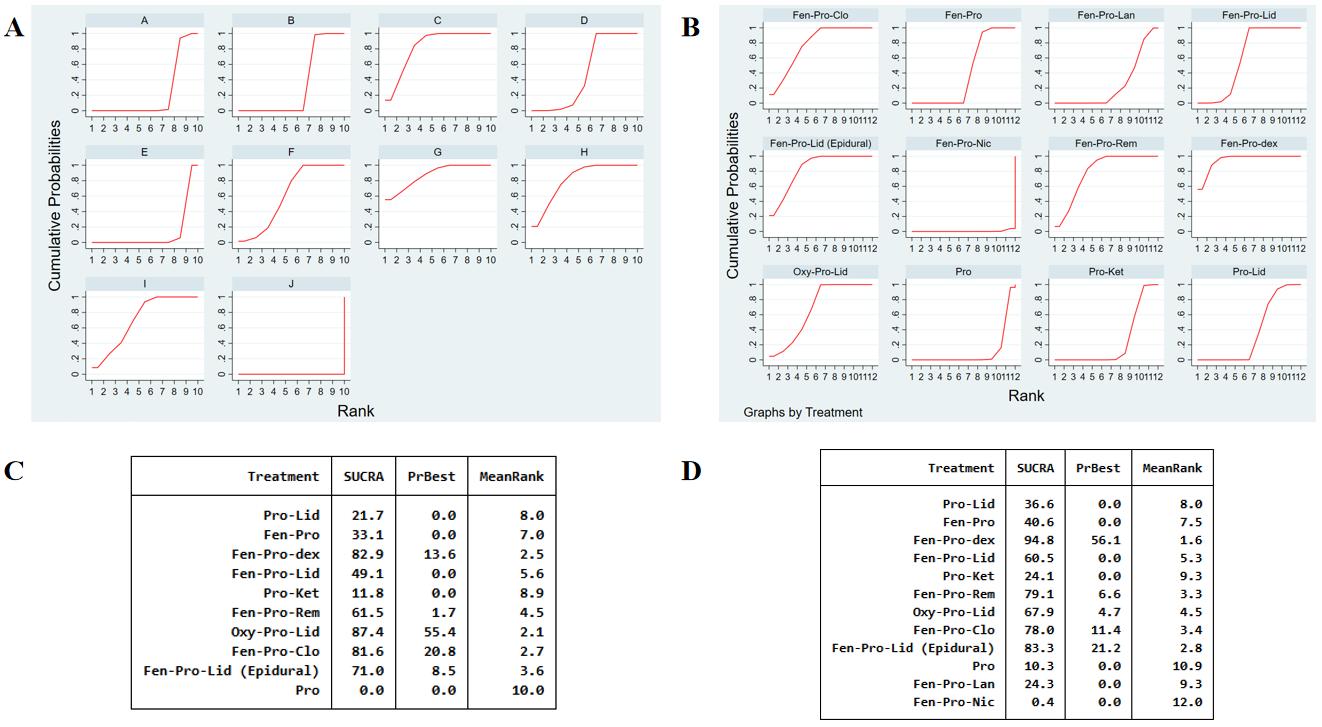

Supplement: Supplementary file 4 [file Image_3.jpg]
